# Complete Genome Sequence of “*Candidatus* Phytoplasma aurantifolia” TB2022, a Plant Pathogen Associated with Sweet Potato Little Leaf Disease in China

**DOI:** 10.1128/mra.00308-23

**Published:** 2023-06-07

**Authors:** Yalu Li, Xiao-Hua Yan, Yanzhi Liu, Shen-Chian Pei, Chih-Horng Kuo, Weijie Huang

**Affiliations:** a Shanghai Center for Plant Stress Biology, CAS Center for Excellence in Molecular Plant Sciences, Chinese Academy of Sciences, Shanghai, China; b Institute of Plant and Microbial Biology, Academia Sinica, Taipei, Taiwan; University of Arizona

## Abstract

The complete genome sequence of “*Candidatus* Phytoplasma aurantifolia” TB2022, which consists of one 670,073-bp circular chromosome, is presented in this work. This bacterium is associated with sweet potato little leaf disease in Fujian Province, China.

## ANNOUNCEMENT

Phytoplasmas are a group of phloem-inhabiting bacteria transmitted by sap-feeding insects (1). They have a broad host range and are associated with a variety of symptoms, such as witches’ broom, little leaf, and phyllody, causing significant damages in many crops. One species-level taxon, “*Candidatus* Phytoplasma aurantifolia,” is widespread in the Western Pacific and has been a persistent threat to sweet potato production in the coastal area of Fujian Province, China, since the 1960s (2). To improve our understanding of this pathogen, we conducted whole-genome shotgun sequencing of a field sample. All kits were used according to the manufacturer’s protocols, and all bioinformatics tools were used with default settings unless stated otherwise.

Strain TB2022 was collected from symptomatic sweet potatoes cultivated in Tianbian Village (Hui’an County, Fujian Province) in July 2022. Leaves from one plant exhibiting severe witches’ broom and little leaf symptoms were collected for DNA extraction using the Hi-DNAsecure plant kit (DP350; Tiangen Biotech). For Illumina sequencing, a library was prepared using the VAHTS universal Plus DNA library prep kit (ND617-C3-02; Vazyme), followed by sequencing on the Illumina NovaSeq 6000 platform to generate ~66.8 million reads, totaling 10.0 Gb. For Oxford Nanopore Technologies (ONT) sequencing, DNA fragments of >10 kb were collected using the BluePippin system (Sage Science), followed by processing using NEBNext formalin-fixed, paraffin-embedded (FFPE) DNA repair mix and the NEBNext Ultra II end repair/dA-tailing module (New England Biolabs). A library was prepared using the ONT ligation sequencing kit (SQK-LSK109) without shearing and sequenced using PromethION flow cells (FLO-PRO002) on the PromethION 48 system. Guppy v3.2.6 was used for base calling with fast mode, which produced 799,539 reads totaling 12.1 Gb (*N*_50_, 25.4 kb).

By mapping the Illumina reads to published phytoplasma genomes (3) using BWA v0.7.17 (4), TB2022 was found to have ~99% genome-wide average nucleotide identity with “*Ca.* Phytoplasma aurantifolia” NCHU2014 (5, 6) (GenBank accession number CP040925). Thus, a two-step assembly procedure was utilized. First, the Illumina and ONT reads were mapped to the reference genome using BWA v0.7.17 (4) and Minimap2 v2.15 (7), respectively. Mapped reads with an alignment score above 30 (for Illumina) or 1,000 (for ONT) were extracted and used for assembly with Trycycler v0.5.3 (8). The gene prediction and annotation procedure was based on the one described in our previous work (9, 10). For gene prediction, RNAmmer v1.2 (11), tRNAscan-SE v1.3.1 (12), and Prodigal v2.6.3 (13) were used. Annotation was performed based on the homologs in other phytoplasmas, as identified using OrthoMCL v1.3 (14), followed by manual curation using BlastKOALA v12 (15) and GenBank (16). Putative secreted proteins were identified using SignalP v5.0 (17) and filtered using TMHMM v2.0 (18).

The reference-based assembly produced one circular chromosome of 670,073 bp, with 25.0% G+C content; no plasmids were found. The average sequencing depth is 885-fold, based on ~4.4 million Illumina reads, and 618-fold, based on 71,436 ONT reads (*N*_50_, 21.0 kb). The contig was rotated to have *dnaA* as the first gene. The annotation contains 6 rRNA genes, 27 tRNA genes, 535 protein-coding genes, and 22 pseudogenes.

### Data availability.

This genome project has been deposited at NCBI under the BioProject accession number PRJNA937424. All raw reads derived from the infected plant sample have been deposited at the NCBI Sequence Read Archive under the accession numbers SRX19479157 and SRX19479158. The phytoplasma genome sequence has been deposited at GenBank under the accession number CP120449.
